# Supplementary material for: Heat adaptation of phage T7 under an extended genetic code
Source: Virus Evol. 2021 Dec 1;7(2):veab100. doi: 10.1093/ve/veab100 (PMC8923235; doi:10.1093/ve/veab100)
Supplement: veab100_Supp [file veab100_supp.zip › Sequencing_Analysis.zip!/Sequencing_Analysis/A3/evidence/JC_SIDE_1_75.html]

BRESEQ :: Evidence


| New junction evidence | | | | | | | | | | | |
| --- | --- | --- | --- | --- | --- | --- | --- | --- | --- | --- | --- |
|  | | seq id | position | reads (cov) | reads (cov) | score | skew | freq | annotation | gene | product |
| \* | ? | NC\_001604.1 | = 19635 | 393 (0.230) | 1117 (0.650) | 309/590 | 0.2 | 74.6% | intergenic (–/–) | *–/–* | –/– |
|  | ? | NC\_001604.1 | 19645 = | 374 (0.220) |  |  |  |  | intergenic (–/–) | *–/–* | –/– |

Only 100 of 1530 total aligned reads displayed.  

`AAGCTGTGACATTCACCACAAAGGAGACATTCTGGATGTAACGTCCGTTGTCATTAATCCTGAGGCAGACTCTAAGGGCTTACAGCGATTCCTAGCGAAACGCTTTAAGTACCTTGCGGAACTCCACGATTGCGATTGGGTGTCTCGTTGTAAGCATGAAGGCGAGACAATGCGTGTATACTTTAAGGAGGTATAAGTTATGGGTAAGAAAGTTAAGAAGGCCGTGAAGAAAGTCACCAAGTCCGTTAAGAAAGTCGTTAAGGAAGGGGCTCGTCCGGTTAAACAGGTTGCTGGCGGTCTAGCTGGTCTGGCTGGTGGTACTGGTGAAGCACAGATGGTGGAAGTACCACAAGCTGCCGCACAGATTGTTGACGTACCTGAGAAAGAGGTTTCCACTGAGGACGAAGCACAGACAGAAAGCGGACGCAAGAAAGCTCGTGCTGGCGGTAAGAAATCCTTGAGTGTAGCCCGTAGCTCCGGTGGCGGTATCAACATTTAATCAGGAGGTTATCGTGGAAGACTGCATTGAATGGACCGGAGGTGTCAACTCTAAGGGTTATGGTCGTAAGTGGGTTAATGGTAAACTTGTGACTCCACATA  >  NC_001604.1/19336‑19935`  

                                                                                                                                                                                                                                                                                                           `|`                                                                                                                                                                                                                                                                                                              
   `tataagagcccgccacaaaggagacattCTGGATGTAACTTCCGTTGGCATTAATCCTGAGGCAGACTCTAAGGGCTTACAGCGATTCCTAGCGAAACGCTTTAAGTACCTTGCGGAACTCCACGATTGCGATTGGGTGTCTCGTTGTAAGCATGAAGGCGAGACAATGCGTGTATACTTTAAGGAGGTATAAGTTATGGGTAAGAAAGTTAAGAAGGCCGTGAAGAAAGTCACAAAGTCCGTTAAGAAAGTCGTTAAGGAAGGGGCTCGTCCGGTTAAACAGGTTGCTGGCGGTCTggct                                                                                                                                                                                                                                                                                                          <  1:8332‑M1/289‑5 (MQ=255)`  
     `gtgACATTCACCACAAAGGAGACATTCTGGATGTAACGTCCGTTGTCATTAATCCTGAGGCAGACTCTAAGGGCTTACAGCGATTCCTAGCGAAACGCTTTAAGTACCTTGCGGAACTCCACGATTGCGATTGGGTGTCTCGTTGTAAGCATGAAGGCGAGACAATGCGTGTATACTTTAAGGAGGTATAAGTTATGGGTAAGAAAGTTAAGAAGGCCGTGAAGAAAGTCACCAAGTCCGTTAAGAAAGTCGTTAAGGAAGGGGCTCGTCCGGTTAAACAGGTTGCTGGCGGTCTggctgg                                                                                                                                                                                                                                                                                                        <  1:262672‑M1/301‑7 (MQ=255)`  
     `gtgACATTCACCACAAAGGAGACATTCTGGATGTAACGTCCGTTGTCATTAATCCTGAGGCAGACTCTAAGGGCTTACAGCGATTCCTAGCGAAACGCTTTAAGTACCTTGCGGAACTCCACGATTGCGATTGGGTGTCTCGTTGTAAGCATGAAGGCGAGACAATGCGTGTATACTTTAAGGAGGTATAAGTTATGGGTAAGAAAGTTAAGAAGGCCGTGAAGAAAGTCACCAAGTCCGTTAAGAAAGACGTTAAGGAAGGGGCTCGTCAGGTTAAACAGGTTGCTGGCGGTCTgactgg                                                                                                                                                                                                                                                                                                        >  1:281065‑M1/1‑295 (MQ=255)`  
     `gagatgtgtataagagacaggaCATTCTGGATGTAACGTCCGTTGTCATTAATCCTGAGGCAGACTCTAAGGGCTTACAGCGATTCCTAGCGAAACGCTTTAAGTACCTTGCGGAACTCCACGATTGCGATTGGGTGTCTCGTTTTAAGCATGAAGGCGAGGCAATGCGTGTATACTTTAAGGAGGTATAAGTTATGGGTAAGAAAGTTAAGAAGGCCGTGAAGAAAGACACCAAGTCCGTTAAGAAAGTCGTTAAGGAAGGGGCTCGTCCGGTTAAACAGGTTTCTGGCGGTCTAGCTgg                                                                                                                                                                                                                                                                                                        <  1:15152/281‑1 (MQ=255)`  
        `aCATTCACCACAAAGGAGACAGTCTGGATGTAACGTCCGTTGTCATTAATCCTGAGGCATACTCTAAGGGCTTACAGCGATTCCTAGCGAAACGCTTTAAGTACCTTGCGGAACTCCACGATTGCGATTGGGTGTCTCGTTGTAAGCATGAAGGCGAGACAATGCGTGTATACTTTAAGGAGGTATAAGTTATGGGTAAGAAAGTTAAGAAGGCCGTGAAGAAAGTCACCAAGTCCGTTAAGAAAGTCGTTAAGGAAGGGGCTCGTCCGGTTAAACAGGTTGCTGGCGGTCTggctggtgg                                                                                                                                                                                                                                                                                                     <  1:283506‑M1/301‑10 (MQ=255)`  
          `cttttccatgaaggttccATTCTGTATGTAACGTCTGTTGTCACTAATTATGAGGCAGACTATCAGGGCTTTCAGCGCTTCTTAGCCAATCTCTTTAAGTACCTCTCGGAATTCCCCGATTGCTATTGGCTGTCTTGTTGTAAGCATGAAGGAGAGACAATGCTTGTATACTTTAAGGAGGTGTAATTTATGGGTAAGAAAGTTAAGAAGGCCGTGAAGAAAGTCTCCTAGTCCGTTAAGAATGTCTTTTAGGAAGGGGCTCGTCCGGTTAAACAGGTTGCTGGCGGTCTGGCTGGtggta                                                                                                                                                                                                                                                                                                   <  1:145738/284‑5 (MQ=255)`  
          `aTTCACCACAAAGGAGACATTCTGGATGTAACGTCCGTTGTCATTAATCCTGAGGCAGACTCTAAGGGCTTACAGTGATTCCTAGCGAAACGCTTTAAGTACCTTGCGGAACTCCACGATTGCGATTGGGTGTCTCGTTGTAAGCATGAAGGCGAGACAATGCGTGTATACTTTAAGGAGGTATAAGTTATGGGTAAGAAAGTTAAGAAGGCCGTGAAGAAAGTCACCAAGTCCGTTAAGAAAGTCGTTAAGGAAGGGGCTCGTCCGGTTAAACAGGTTTCTGGCGGTCTAGCTGGTctgg                                                                                                                                                                                                                                                                                                   >  1:334600/1‑301 (MQ=255)`  
          `aTTCACCACAAAGGAGACATTCTGGATGTAACGTCCGTTGTCATTAATCCTGAGGCAGACTCTAAGGGCTTACAGCGATTCCTAGCGAAACGCTTTAAGTACCTTGCGGAACTCCACGATTGCGATTGGGTGTCTCGTTGTAAGCATGAAGGCGAGACAATGCGTGTATACTTTAAGGAGGTATAAGTTATGGGTAAGAAAGTTAAGAAGTCCGTGAAGAAAGTCACCAAGTCCGTTAAGAAAGTCGTTAAGGAAGGGGCTCGTCCGGTTAAACAGGTTGCTGGCGGTCTggctggtggta                                                                                                                                                                                                                                                                                                   >  1:255170‑M1/1‑290 (MQ=255)`  
           `ttCACCACAAAGGAGACATTCTGGATGTAACGTCCGTTGGCATTAATCCTGAGGCAGACTCTAAGGGCTTACAGCGATTCCTAGCGAAACGCTTTAAGTACCTTGCGGAACACCACGATTGCGATTGGGTGTCTCGTTGTAAGCATGAAGGCGAGACAATGCGTGTATACTTTAAGGAGGTATAAGTTATGGGTAAGAAAGTTAAGAAGGCCGTGAAGAAAGTCACCAAGTCCGTTAAGAAAGTCGTTAAGGAAGGGGCTCGTCCGGTTAAACAGGTTGCTGGCGGTCTggctggtagtac                                                                                                                                                                                                                                                                                                  <  1:161879‑M1/301‑13 (MQ=255)`  
                 `acaAAGTAGACATTATGGATGTAACTGCCGTTGTCATTAAGCCGAAGGCAGAATATAAGGGCGTACAGCGATTACTAGAGAAACGCGTGAAGTACCTTGCGGAACTCCACGATTGCGATTGGGTGTCTCGTCGTAAGCATGAAGGCGAGACAATGCGTGTATACTTTATGGTGGTATAAGTTATGGGTAAGAAAGTTAAGAAGGCCGTGAAGAAAGTCACCAAGTCCGTTAAGAAAGTCGTTAAGGAAGGGGCTCGTCCGGTTAAACAGGTTGCTGGCGGTCTagctggtggtactggtga                                                                                                                                                                                                                                                                                            <  1:80521‑M1/301‑19 (MQ=255)`  
                  `caAAGGAGACATTCTGGATGTAACGTCCGTTGTCATTAATCCTGAGGCAGACTCTAAGGGCTTACAGCGATTCCTAGCGAAACGCTTTAAGTACCTTGCGGAACTCCACGATTGCGATTGGGTGTCTCGTTGTAAGCATGAAGGCGAGACAATGCGTGTATACTTTAAGGAGGTATAAGTTATGGGTAAGAAAGTTAAGAAGGCCGTGAAGAAAGTCACCAAGTCCGTTAAGAAAGTCGTTAAGGAAGGGGCTCGTCCGGGTAAACAGGTTGCTGGCGGTCTggctgggggtactggtgaa                                                                                                                                                                                                                                                                                           >  1:200850‑M1/1‑282 (MQ=255)`  
                      `ggAGACATTCTGGATGTAACGTCCGTTGTCATTAATCCTGAGGCAGACTCTAAGGGCTTACAGCGATTCCTAGCGAAACGCTTTAAGTACCTTGCGGAACTCCACGATTGCGATTGGGTGTCTCGTTGTAAGCATGAAGTCGAGACAATGCGTGTATACTTTAAGGAGGTATAAGTTATGGGTAAGAAAGTTAAGAAGGCCGTGAAGAAAGTCACCAAGTCCGTTAAGAAAGTCGTTAATGAAGGGGCTCGTCCGGTTAAACAGGTTGCTGGCGGTCTggctggtggtactggtgaagcac                                                                                                                                                                                                                                                                                       >  1:42415‑M1/1‑278 (MQ=255)`  
                        `agaCATTCTGGATGTAACGTCCGTTGTCATTAATCCTGAGGCAGACTCTAAGGGCTTACAGCGATTCCTAGCGAAACGCTTTAAGTACCTTGCGGAACTCCACGATTGCGATTGGGTGTCTCGTTGTAAGCATGAAGGCGAGACAATGCGTGTATACTTTAAGGAGGTATAAGTTATGGGTAAGAAAGTTAAGAAGGCCGTGAAGAAAGTCACCAAGTCCGTTAAGAAAGTCGTTAAGGAAGGGGCTCGTCCGGTTAAACAGGTTGCTGGCGGTCTggctggtggtactggtgaagcacag                                                                                                                                                                                                                                                                                     >  1:203753‑M1/1‑276 (MQ=255)`  
                             `ttCTGGATGTAACGTCCGTTGTCATTAATCCTGAGGCAGACTCTAAGGGCTTACAGCGATTCCTAGCGAAACGCTTTAAGTACCTTGCGGAACTCCACGATTGCGATTGGGTGTCTCGTTGTAAGCATGAAGGCGAGACAATGCGTGTATACTTTAAGGAGGTATAAGTTATGGGTAAGAAAGTTAAGAAGGCCGTGAAGAAAGTCACCAAGTCCGTTAAGAAAGTCGTTAAGGAAGGGGCTCGTCCGGTTAAACAGGTTTCTGGCGGTCTAGCTGGTCTGGCTGGTGGTActgctgtctc                                                                                                                                                                                                                                                                                >  1:295741/1‑297 (MQ=255)`  
                                `tGGTTGTAACGTCCGTTGTAATTAATCCTGAGGCAGACTCTAAGGGCTTACAGCGATTCCTAGCGAAACGCTTTAAGTACCTTGCGGACCTCCACGATTGCGATTGGGTGTCTCGTTGTAAGCATGAAGGCGAGACAATGCGTGTATACTTTAAGGAGGTATAAGTTATGGGTAAGAAAGTTAAGAAGGCCGTGAAGAAAGTCACCAAGTCCGTTAAGAAAGTCGTTAAGGAAGGGGCTCGTCCGGTTAAACAGGTTGCTGGCGGTCTggctggtggtactggtgaagcacagatggtgga                                                                                                                                                                                                                                                                             <  1:22272‑M1/301‑34 (MQ=255)`  
                                `gggaTGGAACGTCCGTTGTCATTAATCCTGAGGCAGACTCTAAGGGCTTAAAGCGATTCCTAGCGAAACGCTTTAAGTACCTTGCGGAACTCCACGATTGCGATTGGGTGTCTCGTTGTAAGCATGAAGGCGAGACAATGCGTGTATACTTTAAGGAGGTATAAGTTATGGGTAAGAAAGTTAAGAAGGCCGTGAAGAAAGTCACCAAGTCCGTTAAGAAAGTCGTTAAGGAAGGGGCTCGTCCGGTTAAACAGGTTGCTGGCTGTCTggctggtggtactggtgaagcacagatggtgga                                                                                                                                                                                                                                                                             <  1:71733‑M1/300‑34 (MQ=255)`  
                                `gctcggagatgtgtataagagacagtcctgaggcagactctaagggcttacAGCGATTCCTAGCGAAACGCTTTAAGTACCTTGCGGAACTCCACGATTGCGATTGGGTGTCTCGTTGTAAGCATGAAGGCGAGACAATGCGTGTATACTTTAAGGAGGTATAAGTTATGGGTAAGAAAGTTAAGAAGGCCGTGAAGAAAGTCACAAAGTCCGTTAAGAAAGTCGTTAAGGAAGGGGCTCGTCCGGTTAAACAGGTTGCTGGCGGTCTggctggtggtactggtgaagcacagatggtgga                                                                                                                                                                                                                                                                             <  1:30586‑M1/276‑34 (MQ=255)`  
                                 `ggATGTAACGTCCGTTGTCATTAATCATGAGGCAGACTCTAAGGGCTTACAGCGATTCCTAGCGAAACGCTTTAAGTACCTTGCGGAACTCCACGATTGCGATTGGGTGTCTCGTTGTAAGCATGAAGGCGACACAATGCGTGTATACTTTAAGGAGGTATAAGTTATGGGTAAGAAAGTTAAGAAGGCCGTGAAGAAAGTCACCAAGTCCGTTAAGAAAGTCGTTAAGGAAGGGGCTCGTCCGGTTAAACAGGTTGCTGGCGGTCTggctggtggtactggtgaagcacagatggtggaa                                                                                                                                                                                                                                                                            >  1:177820‑M1/1‑267 (MQ=255)`  
                                   `aTGTAACGTCCGTTGTCATTAATCCTGAGGCAGACTCTAAGGGCTTACAGCGATTCCTAGCGAAACGCTTTAAGTACCTTGCGGAACTCCACGATTGCGATTGGGTGTCTCGTTGTAAGCATGAAGGCGAGACAATGCGTGTATACTTTAAGGAGGTATAAGTTATGGGTAAGAAAGTTAAGAAGGCCGTGAAGAAAGTCACCAAGTCCGTTAAGAAAGTCGTTAAGGAAGGGGCTCGTCCGGTTAAACAGGTTGCTGGCGGTCTggctggtggtactggtgaagcacggatggtggaagt                                                                                                                                                                                                                                                                          >  1:315131‑M1/1‑265 (MQ=255)`  
                                        `aCGTCCGTTGTCATTAATCCTGAGGCAGACTCTAAGGGCTTACAGCGATTCCTAGCGAAACGATTTAAGTACCTTGCGGAACGCCACGATTGCGATTGGGTGTCTCGTTGTAAGCATGAAGGCGAGACAATGCGTGTATACTTTAAGGAGGTATAAGTTATGGGTAAGAAAGTTAAGAAGGCCGTGAAGAAAGTCACCAAGTCCGTTAAGAAAGTCGTTAAGGAAGGGGCTCGTCCGGTTAAACAGGTTGCTGGCGGTCTggctggtggtactggtgaagcacagatggtggaagtaccac                                                                                                                                                                                                                                                                     <  1:74410‑M1/301‑42 (MQ=255)`  
                                                 `gTCATTAATCCTGAGGCAGACTCTAAGGGCTTACAGCGATTCCTAGCGAAACGCTTTAAGTACATTGCGGAACTCCACGATTGCGATTGGGTGTCTCGTTGTAAGCATGAAGGCGAGACAATGCGTGTATACTTTAAGGAGGTATAAGTTATGGGTAAGAAAGTTAAGAAGGCCGTGAAGAAAGTCACCAAGTCCGTTAAGAAAGTCGTTAAGGAAGGGGCTCGTCCGGTTAAACAGGTTGCTGGCGGTCTAGCTGGTCTGGCTGGTGGTACTGGTGAAGCACAGATGGTGGAAGTACcac                                                                                                                                                                                                                                                            <  1:227225/301‑1 (MQ=255)`  
                                                   `cATTAATCCTGAGGCAGACTCTAAGGGCTTACAGCGATTCCTAGCGAAACGCTTTAAGTACCTTGCGGAACTCCACGATTGCGATTTGGTGTCTCGTTGTAAGCATGAAGGCGAGACTCTGCGTGTATACTTTAAGGAGGTATCAGTTCTGGGTAAGAAAGTTAAGAAGGCCGTGAAGCAAGCCACCAATTCCGTTAAGAAAGTCGTTAAGGAAGGGGCTTGTCCGGTTAAACAGGTTGCTGGCGTTTTtgctggtggtactggtgaacctcagatggtggacgtaccacaagctgactct                                                                                                                                                                                                                                                          >  1:171991‑M1/1‑249 (MQ=255)`  
                                                   `cATTAATCCTGAGGCAGACTCTAAGGGCTTACAGCGATTCCTAGCGAAACGCTTTAAGTACCTTGCGGAACTCCACGATTGCGATTGGGTGTCTCGTTGTAAGCATGAAGGCGAGACAATGCGTGTATACTTTAAGGAGGTATAAGTTATGGGTAAGAAAGTTAAGAAGGCCGTGAAGAAAGTCACCAAGTCCGTTAAGAAAGTCGTTAAGGAAGGGGCTCGTCCGGTTAAACAGGTTGCTGGCGGTCTggctggtggtactggtgaagcacagatggtggaagtaccacaagctgccgca                                                                                                                                                                                                                                                          >  1:129120‑M1/1‑249 (MQ=255)`  
                                                               `ggCCGACTCTAAGGGCTTACAGCGATTCCTAGCGAAACGCTTTAAGTACCTTGCGGAACTCCACGATTGCGATTGGGTGTCTAGTTGTAAGCATGAAGGCGAGACAATGCGTGTATACTTTAAGGAGGTATAAGTTATGGGTAAGAAAGTTAAGAAGGCCGTGAAGAAAGTAACCAAGTCCGTTAAGAAAGTCGTTAAGGAAGGGGCTCGTCCGGTTAAACAGGTTTCTGGCGGTCTAGCTGGTCTGGCTGGTGGTACTGGTGCAGCACAGATGGTGGAAGTACCACAAGCTGCCGCACAg                                                                                                                                                                                                                                              >  1:35593/1‑301 (MQ=255)`  
                                                               `ggCAGACTCTAAGAGCTTACGGCGATTCCTAGCGAAACGCTTTAAGTACCTTGCAGAACTCCACGATTGCGATTGGGTGTCTCGTTGTAAGCATGAAGGCTAGACAATGCGTGTATACTTTAAGGAGGTATAAGTTATGGGTAAGAAAGTTAAGAAGGCCGTGAAGAAAGTCACCAAGTCCGTTAAGAAAGTCGTTAAGGAAGGGGCTCGTCCGGTTAAACAGGTTGCTGGCGGTCTggctggtggtactggtgaagcacagatggtggaagtaccacaagctgccgcacagattgttgac                                                                                                                                                                                                                                              <  1:191015‑M1/301‑65 (MQ=255)`  
                                                                   `gACTCTAAGGGCTTACAGCGATTCCTAGCGAAACGCTTTAAGTACCTTGCGGAACTCCACGATTGCGATTGGGTGTCTCGTTGTAAGCATGAAGGCGAGACAATGCGTGTATACTTTAAGGAGGTATAAGTTATGGGTAAGAAAGTTAAGAAGGCCGTGAAGAAAGTCACCAAGTCCGTTAAGAAAGTCGTTAAGGAAGGGGCTCGTCCGGTTAAAGAGGTTGCTGGCTGTCTggctggtggtactggtgaagcacagatggtggaagtaccacaagctgccgcacagatggttgacgtcc                                                                                                                                                                                                                                          >  1:119857‑M1/1‑233 (MQ=255)`  
                                                                     `ctctAAGGGCTTACATCGATTCCTAGCGAAACGCTTTAAGTACCTTGCGGAACTCCACGATTGCGATTGGGTGTCTCGTTGTAAGCATGAAGGCGAGACAATGCGTGTATACTTTAAGTAGGTATAAGTTATGGGTAAGAAAGTTAAGAAGGCCGTGAAGAAAGTCACCAAGTCCGTTAAGAAAGTCGTTAAGGAAGGGGCTCGTCCGGTTAAACAGGTTGCTGGCGGTCTggctggtggtactggtgaagcacagatggtggaagtaccacaagctgccgcacagattgttgacgctgtc                                                                                                                                                                                                                                        >  1:93777‑M1/1‑231 (MQ=255)`  
                                                                            `ggCTTACAGCGATTCCTAGCGAAACGCTTTAAGTACCTTGCGGAACTCCACGATTGCGATTGGGTGTCTCGTTGTAAGCATGAAGGCGAGACAATGCGTGTATACTTTAAGGAGGTATAAGTTATGGGTAAGAAAGTTAAGAAGGCCGTGAAGAAAGTCACCAAGTCCGTTAAGAAAGTCGTTAAGGAAGGGGCTCGTCCGGTTAAACAGGTTGCTGGCGGTCTggctggtggtactggtgaagcacagatgatggaagtaccacatgatgccgcacagattgttgacgtacctgggaaag                                                                                                                                                                                                                                 >  1:86817‑M1/1‑224 (MQ=255)`  
                                                                                    `gCGATTCCTAGCGAAACGCTTTAAGTACCTTGCGGAACTCCACGATTGCGATTGGGTGTCTCGTTGTAAGCATGAAGGCGAGACAATGCGTGTATACTTTAAGGAGGTATAAGTTATGGGTAAGAAAGTTAAGAAGGCCGTGAAGAAAGTCACCAAGTCCGTTAAGAAAGTCGTTAAGGAAGGGGCTCGTCCGGTTAAACAGGTTTCTGGCGGTCTAGCTGGTCTGGCTGGTGGTACTGGTGAAGCACAGATGGTGGAAGTACCACAAGCTGCCGCACAGATTGTTGACGTACCTGAGaaa                                                                                                                                                                                                                         >  1:240088/1‑301 (MQ=255)`  
                                                                                      `gATTCCTAGAGAAACGCTTTAAGTACCTTGCGGAACTCCACGATTGCGATTGGGTGTCTCGTTGTAAGCATGAAGACGAGACAATGCGTGTATACTTTAAGGAGGTATAAGTTATGGGTAAGAAAGTTAAGAAGGCCGTGAAGAAAGTCACCAAGTCCGTTAAGAAAGTCGTTAAGGAAGGGGCTCGTCCGGTTAAACAGGTTGCTGGCGGTCTggctggtggtactggtgaagcacagatggtggaagtaccacaagctgccgcacagattgttgacgtacctgagaaagaggtttccac                                                                                                                                                                                                                       <  1:89284‑M1/301‑88 (MQ=255)`  
                                                                                                   `aCGCTTTAAGTACCTTGCGGAACTCCACGATTGCGATTGGGTGTCCCGTTGTAAGCATGAAGGCGAGACAATGCGTGTATACTTTAAGGAGGTATAAGTTATGGGTAAGAAAGTTAAGAAGGCCGTGAAGAAAGTCACCAAGTCCGTTAAGAAAGTCGTTAAGGAAGGGGCTCGTCCGGTTAAACAGGTTGCTGGCGGTCTggctggtggtactggtgaagcacagatggtggaagtaccacaagctgccgcacagattgttgacgtacctgagaaagaggctgtctcttatacacatctc                                                                                                                                                                                                          >  1:55072‑M1/1‑201 (MQ=255)`  
                                                                                                    `cGCTTTAAGTACCTTGCGGAACTCCACGATTGCGATTGGGTGTCTCGTTGTAAGCATGAAGGCGAGACAATGGGTGTATACTTTAAGGAGGTATAAGTTATGGGTAAGAAAGTTAAGAAGGCCGTGAAGAAAGTCACCAAGTCCGTTAAGAAAGTCGTTAAGGAAGGGGCTCGTCCGGTTAAACAGGTTGCTGGCGGTCTggctggtggtactggtgaagcacaaatggtggaagtaccacaagctgccgcacagattgttgacgtacctgagaaagaggtttccactgaggacgaagcac                                                                                                                                                                                                         <  1:211708‑M1/301‑102 (MQ=255)`  
                                                                                                    `cGCTTTAAGTACCTTGCGGAACTCCACGATTGCGATTGGGTGTCTCGTTGTAAGCATGAAGGCGAGACAATGCGTGTATACTTTAAGGAGGTATAAGTTATGGGTAAGAAAGTTAAGAAGGCCGTGAAGAAAGTCACCAAGTCCGTTAAGAAAGTCGTTAAGGAAGGGGCTCGTCCGGTTAAACAGGTTGCTGGCGGTCTggctggtggtactggtgaagcacagatggtggaagtaccacaagctgccgcacagattgttgacgtacctgataaagaggtttccactgaggacgaagcac                                                                                                                                                                                                         >  1:64762‑M1/1‑200 (MQ=255)`  
                                                                                                    `cGCTTTAAGTACCTTGCGGAACTCCACGATTGCGATTGGGTGTCTCGTTGTAAGCATGAAGGCGAGACAATGCGTGTATACTTTAAGGAGGTATAAGTTATGGGTAAGAAAGTTAAGAAGGCCGTGAAGAAAGTCACCAAGTCCGTTAAGAAAGTCGTTAAGGAAGGGGCTCGTCCGGTTAAACAGGTTGCTGGCGGTCTggctggtggtactggtgaagcacagatggtggaagtaccacaagctgccgcacagattgttgacgtacctgagaaagaggtttccactgaggacgaagcac                                                                                                                                                                                                         <  1:257454‑M1/301‑102 (MQ=255)`  
                                                                                                     `gCTTTAAGTACCTTGCGGAACTCCACGATTGCGATTGGGTGTCTCGTTGTAAGCATGAAGGCGAGACAATGCGTGTATACTTTAAGGAGGTATAAGTTATGGGTAAGAAAGTTAAGAAGGCCGTGAAGAAAGTCACCAAGTCCGTTAAGAAAGTCGTTAAGGAAGGGGCTCGTCCTGTTAAACAGGTTTCTGGCGGTCTAGCTGGTCTGGCTGGTGGTACTGGTGAAGCACAGATGGTGGAAGTACCACAAGCTGCCGCACAGATTGTTGACGTACCTGAGAAAGAGGTTTCCACTGAGGa                                                                                                                                                                                                        >  1:274647/1‑301 (MQ=255)`  
                                                                                                      `cTTTAAGTACCTTGCGGAACTCCACGATTGCGATTGGGTGTCTCGTTGTAAGCATGAAGGCGAGACAATGCGTGTATACTTTAAGGAGGTATAAGTTATGGGTAAGAAAGTTAAGAAGGCCGTGAAGAAAGTCACCAAGTCCGTTAAGAAAGTCGTTAAGGAAGGGGCTCGTCCGGTTAAACAGGTTGCTGGCGGTCTggctggtggtactggtgaagcacagatggtggaagtaccacaagctgccgcacagattgttgacgtacctgagaaagaggtttccactgcggtctcttataca                                                                                                                                                                                                       >  1:299782‑M1/1‑198 (MQ=255)`  
                                                                                                      `cTTTAAGTACCTTGCGGAACTCCACGATTGCGATTGGGTGTCTCGTTGTAAGCATGAAGGCGAGACAATGCGTGTATACTTTAAGAAGGTATAAGTTATGGGTAAGAAAGTTAAGAAGGCCGTGAAGAAAGTCACCACGTCCGTTAAGAAAGTCGTTAAGGAAGGGGATCGTCCGGTTAAACAGGTTGCTGGCGGTCTggctggtggtactggtgaagcacagatggtggaagtaccacaagctgccgcacagattgttgacgtacctgagaaagaggtttccactgaggacgaagcacag                                                                                                                                                                                                       >  1:143598‑M1/1‑198 (MQ=255)`  
                                                                                                        `ttAAGTACCTTGCGGAACTCCACGATTGCGATTGGGTGTCTCGTTGTAAGCATGAAGGCGAGACAATGCGTGTATACTTTAAGGAGGTATAAGTTATGGGTAAGAAAGTTAAGAAGGCCGTGAAGAAAGTCACCAAGTCCGTTAAGAAAGTCGTTAAGGAAGGGGCTCGTCCGGTTAAACAGGTTGCTGGCGGTCTggctggttgtactggtgaagcacagatggtggaagtaccacaagctgcctcacagattgttgacgtacctgagaaagatgtttccactgaggacgaagcacagac                                                                                                                                                                                                     <  1:340276‑M1/301‑106 (MQ=255)`  
                                                                                                        `ttAAGTACCTTGCGGAACTCCACGATTGCGATTGGGTGTCTCGTTGTAAGCATGAAGGCGAGACAATGCGTGTATACTTTAAGGAGGTATAAGTTATGGGTAAGAAAGTTAAGAAGGCCGTGAAGAAAGTCACCAAGTCCGTTAAGAAAGTCGTTAAGGAAGGGGCTCGTCCGGTTAAACAGGTTGCTGGCGGTCTggctggtggtactggtgaagcacagatggtggaagtaccacaagctgccgcacagattgttgacgtacctgagaaagaggtttccactgaggacgaagcacagac                                                                                                                                                                                                     <  1:123206‑M1/301‑106 (MQ=255)`  
                                                                                                            `ataCCTTGCGGAACTCCACGATTGCGGTTGGGTGTCTCGTTGTAAGCATGAAGGGGAGACAATGCGTGTATACTTTAAGGAGGTATAAGTTATGGGTAAGAAAGTTAAGAAGGCCGTGAAGAAAGTCACCAAGTCCGTTAAGAAAGTCGTTAAGGAAGGGGCTCGTCCGGTAAAACAGGTTGCTGGCGGTCTggctggtggtactggtgaagcacagatggtggaagtaccacaagctgccgcacagattgttgacgtacctgagaaagaggtttccactgaggacgaagcacagacagaa                                                                                                                                                                                                 <  1:105713‑M1/300‑110 (MQ=255)`  
                                                                                                              `aCCTTGCGGAACTCCACGATTGCGATTGGGTGTCTCGTTGTAAGCATGAAGGCGAGACAATGCGTGTATACTTTAAGGAGGTATAAGTTATGGGTAAGAAAGTTAAGAAGGCCGTGAAGAAAGTCACCAAGTCCGTTAAGAAAGTCGTTAAGGAAGGGGCTCGTCCGGTTAAACAGGTTGCTGGCGGTCTggctggtggtactggtgaagcacagatggtggaagtaccacaagctgccgcacagattgttgacgtacctgagaaagaggtttccactgaggacgaagcacagacagaaag                                                                                                                                                                                               <  1:158402‑M1/301‑112 (MQ=255)`  
                                                                                                                 `gtGCGGAACTCAACGAGTGGGATTGGGTGGATGGTTGTAAGAATGAAGGAGAGACAATGCGTGTATACTTTAAGGAGGTATAAGTTAGGGGTAAGAAAGTTAAGAAGGCCGTGAAGAAAGTCACCAAGGCCGTTAAGAAAGTCGTTAAGGAAGGGGCTCGTCCGGTTAAACAGGTTGCTGGCGGTCTAGCTGGTCTGGCTGGTGGTACTGGTGAAGCACAGATGGTGGAAGTACCACAAGCTGCCGCACAGATTGTTGACGTACCTGAGAAAGAGGTTTCCACTGAGGACGAAGCacagac                                                                                                                                                                                            <  1:337500/300‑1 (MQ=255)`  
                                                                                                                        `aCTCCACGATTGCGATTGGGTGTCTCGTTGTAAGCATGAAGGCGAGACAATGCGTGTATACTTTAAGGAGGTATAAGTTATGGGTAAGAAAGTTAAGAAGGCCGTGAAGAAAGTCACCAAGTCCGTTAAGAAAGTCGTTAAGGAAGGGGCTCGTCCGGTTAAACAGGTTGCTGGCGGTCTggctggtggtactggtgaagcacagatggtggaagtaccacaagctgccgcacagattgttgacgtacctgagaaagaggtttccactgaggacgaagcacagacagaaagcggacgcaag                                                                                                                                                                                     >  1:51852‑M1/1‑180 (MQ=255)`  
                                                                                                                         `cTCCACGATTGCGATTGGGTGTCTCGTTGTAAGCATGAAGGCGAGACAATGCGTGTATACTTTAAGGAGGTATAAGTTATGGGTAAGAAAGTTAAGAAGGCCGTGAAGAAAGTCACCAAGTCCGTTAAGAAAGTCGTTAAGGAAGGGTCTCGTCCGGTTAAACAGGTTTCTGGCGGTCTAGCTGGTCTGGCTGGTGTTACTGGTGAAGCACAGATGGTGGAAGTACCACAAGCTGCCGCACAGATTGTTGACGTACCAGAGAAAGAGGTTTCCACTGAGGACGAAGCAAAGACAGAAAgag                                                                                                                                                                                    >  1:326173/1‑299 (MQ=255)`  
                                                                                                                                         `ggggGTCTGGTTGTAAGCATGAAGGCGAGACAATGCGTGTATACTTTAAGGAGGTATAAGTTATGGGTAAGAAAGTTAAGAAGGCCGTGAAGAAAGTCACCAAGTCCGTTAAGAAAGTCGTTAAGGAAGGGGCTCGTCCGGTTAAACAGGTTGCTGGCGGTCTggctggtggtactggtgaagcacagatggtggaagtaccacaagctgccgcacagattgttgacgtacctgagaaagaggtttccactgaggacgaagcacagacagaaagcggacgcaagaaagctcgtgctggcgg                                                                                                                                                                    <  1:23677‑M1/301‑139 (MQ=255)`  
                                                                                                                                             `gtCTCGTTGTAAGCATGAAGGCGAGACAATGCGTGTATACTTTAAGGAGGTATAAGTTATGGGTAAGAAAGTTAAGAAGGACGTGAAGAAAGTCACCAAGTCCGTTAAGAAAGTCGTTAAGGAAGGGGCTCGTCCGGTTAAACAGGTTGCTGGCGGTCTggctggtggtactggtgaagcacagatggtggcagtaccacaagctgccgcacagattgttgacgtacctgagaaagaggtttccactgaggacgaagcacagacagaaagcggacgcaagaaagctcgtgctggcggtaag                                                                                                                                                                >  1:248694‑M1/1‑159 (MQ=255)`  
                                                                                                                                             `aaacagttgtaAGCAGGAAGGCGAGACAAAGCGTGTATACTTTAAGGAGGGATAAGTGATGGGTAAGAAAGTTAAGAAGGCCGTGAAGAAAGTCACCAAGTCCGTTAAGAAAGTCGTTAAGGAAGGGGCTCGTCCGGTAAAACAGGTTGCTGGCGGTCTggctggtggtactggtgaagcacagatggtggaagtaccacaagctgccgcacagattgttgacgtacctgagaaagaggtttccactgaggacgaagcacagacagaaagcggacgcaagaaagctcgtgctggcggtaag                                                                                                                                                                <  1:154145‑M1/296‑143 (MQ=255)`  
                                                                                                                                                      `tAAGCATGAAGGCGAGAAAATTCGTGTATACTTGAAGGAGGTATAAGTTATGGGTAAGAAAGTTAAGAAGGCCGTGAAGAAAGTCACCAAGTCCGTTAAGAAAGTCGTTAAGGAAGGGGCTCGTCCGGTTAAACAGGTTGCTGGCGGTCTagctggtggtactggtgaagcacagatggtggaagtaccacaagctgccgcacagattgttgacgtacctgagaaagaggtttccactgaggacgaagcacagacagaaagcggacgcaagaaagctcgtgctggcggtaagaaatccttg                                                                                                                                                       <  1:131115‑M1/301‑152 (MQ=255)`  
                                                                                                                                                      `aaagCATGAAGGCGAGACAATGGGTGTATACTTTAAGGAGGTATAAGTTATGGGTAAGAAAGTTAAGAAGGCCGTGAAGAAAGTCACAAAGTCCGTTAAGAAAGTCGTTAAGGAAGGGGCTCGTCCGGTTAAACAGGTTGCTGGCGGTCTggctggtggtactggtgaagcacagatggtggaagtaccacaagctgccgcacagattgttgacgtacctgagaaagaggtttccactgaggacgaagcacagacagaaagcggacgcaagaaagctcgtgctggcggtaagaaatccttg                                                                                                                                                       <  1:206940‑M1/300‑152 (MQ=255)`  
                                                                                                                                                         `gCATGAAGGCGAGACAATGCGTGTATACTTTAAGGAGGTATAAGTTATGGGTAAGAAAGTTAAGAAGGCCGTGAAGAAAGTCACCAAGTCCGTTAAGAAAGTCGTTAAGGAAGGGGCTCGTCCGGTTAAACAGGTTGCTGGCGGTCTggctggtggtactggtgaagcacagatggtggaagtaccacaagctgccgcacagattgttgacgtacctgagaaagaggtttccactgaggacgaagcacagacagaaagcggacgcaagaaagctcgtgcttgcggtaagaaatccttgagg                                                                                                                                                    >  1:233587‑M1/1‑147 (MQ=255)`  
                                                                                                                                                            `atgaGGCGAGACAATGCGTGTATACTTTAAGGAGGTATAAGTTATGGGTAAGAAAGTTAAGAAGGCCGTGAAGCAAGTCACCAAGTCCGTTAAGAAAGTCGTTAAGGAAGGGGCTCGTCCGGTTAAACAGGTTTATGGCGGTCTAGCTGGTCTGGCTGGTGGTACTGGTGAAGCACAGATGGTGGAAGTACCACAAGCTGCCGCACAGATTGTTGACGTACCTGAGAAAGAGGTTTCCACTGAGGACGAAGCACAGACAGAAAGCGGACGCAAGAAAGCTCGTGCTGGCGGTAAGAAATcc                                                                                                                                                 <  1:126506/298‑1 (MQ=255)`  
                                                                                                                                                              `aaGGCGAGACAATGCGTGTATACTTTAAGGAGGTATAAGTTATGGGTAAGAAAGTTAAGAAGGCCGTGAAGAAAGTCACCAAGTCCGTTAAGAAAGTCGTTAAGGAAGGGGCTCGTCCGGTTAAACAGGTTGCTGGCGGTCTggctggtggtactggtgaagcacagatggtggaagtaccacaagctgccgcacagattgttgacgtacctgagaaagaggtttccactgaggacgaagcacagacagaaagcggacgcaagaaagctcgtgctggcggtaagaaatccttgagtgtagc                                                                                                                                               >  1:67275‑M1/1‑142 (MQ=255)`  
                                                                                                                                                               `aGGCGGGACAATGCAGGTATACTTTAAGGAGTTATAAGTTATGGGTAAGAAAGTTAAGAAGGCCGTGAAGAAAGTCACCAAGTCCGTTAAGAAAGTCGTTAAGGAAGGGGCTCGTCCGGTTAAACAGGTTGCTGGCGGTCTggctggtggtactggtgaagcacagatggtggaagtaccacaagctgccgcacagattgttgacgtacctgagaaagaggtttccactgaggacgaagcacagacagaaagcggacgcaagaaagctcgtgctggcggtaagaaatccttgagtgtagcc                                                                                                                                              <  1:320105‑M1/301‑161 (MQ=255)`  
                                                                                                                                                               `aGGCGAGACAATGCGTGTATACTTTAAGGAGGTATAAGTTATGGGTAAGAAAGTTAAGAAGGCCGTGAAGAAAGTCACCAAGTACGTTAAGAAAGTCGTTAAGGAAGGGGCTCGTCCGGTTAAACAGGTTTCTGGCGGTCTAGCTGGTCTGGCTGGTGGTACTGGTGAAGCACAGATGGTGGAAGTACCACAAGCTGCCGCACAGATTGTTGACGTACCTGAGAAAGAGGTTACCACTGAGGACGAAGCACAGACAGAAAGCGGACGCAAGAAAGCTCGTGCTGGCGGTAAGAAATCCTTg                                                                                                                                              >  1:107342/1‑301 (MQ=255)`  
                                                                                                                                                                `ggCGAGACAATGCGTGTATACTTTAAGGAGGTATAAGTTATGGGTAAGAAAGTTAAGAAGGCCGTGAAGAAAGTCACCAAGTCCGTTAAGAAAGTCGTTAAGGAAGGGGCTCGTCCGGTTAAACAGGTTTCTGGCTGTCTAGCTGGTCTGGCTGGTGGTACTGGTGAAGCACAGATGGTGGAAGTACCACAAGCTGCCGCACAGATTTTTGACGTACCTGAGAAAGAGGTTTCCACTGAGGACGAAGCACAGACAGAAAGCGGACGCAAGCAAGCTCGTGCTGGCGGTAAGAACTCCTTGa                                                                                                                                             >  1:181945/1‑301 (MQ=255)`  
                                                                                                                                                                `ggCGAGACAATGCGTGTATACTTTAAGGAGGTATAAGTTATGGGTAAGAAAGTTAAGAAGGCCGTGAAGAAAGTCACCAAGTCCGTTAAGAAAGTCGTTAAGGAAGGGGCTCGTCCGGTTAAACAGGTTGCTGGCGGTCTggctggtggtactggtgaagcacagatggtggaagtaccacaagctgccgcacagattgttgacgtacctgggaaagaggtttccactgaggacgaagcacagacagaaagcggacgcaagaaagctcgtgctggcggtaagaaaaccttgagtgtagccc                                                                                                                                             >  1:59162‑M1/1‑140 (MQ=255)`  
                                                                                                                                                                   `gagaCAATGCGTGTATACTTTAAGGAGGTATAAGTTATGGGTAAGAAAGTTAAGAAGGCCGTGAAGAAAGTCACCAAGTCCGTTAAGAAAGTCGTTAAGGAAGGGGCTCGTCCGGTTAAACAGGTTTCTGGCGGTCTAGCTGGTCTGGCTGGTGGTACTGGTGAAGCACAGATGGTGGAAGTACCACAAGCTGCCGCACAGATTGTTGACGTACCTGAGAAAGAGGTTTCCACTGAGGACGAAGCACAGACAGAAAGCGGACGCAAGAAAGCTCGTGCTGGCGGTAAGAAATCCTTGAgtg                                                                                                                                          <  1:329680/301‑1 (MQ=255)`  
                                                                                                                                                                           `gCGTGTATACTTTAAGGAGGTATAAGTTATGGGTAAGAAAGTTGAGAAGGCCGTGAAGAAAGTCACCAAGTCCGTTAAGAAAGTCGTTAAGGAAGGGGCTCGTCCGGTTAAACAGGTTGCTGGCGGTCTggctggtggtactggtgaagcacagatggtggaagtaccacaagctgccgcacagattgttgacgtacctgagaaagaggtttccactgaggacgaagcacagacagaaagcggacgcaagaaagctcgtgctggcggtaagaaatccttgagtgtagcccgtagctccggt                                                                                                                                  <  1:116586‑M1/301‑173 (MQ=255)`  
                                                                                                                                                                           `gCGTGTATACTTTAAGGAGGTATAAGTTATGGGTAAGAAAGTTAAGAAGGCCGTGAAGAAAGTCACCAAGTCCGTTAAGAAAGTCGTTAAGGAAGTGGCTCGTCCGGTTAAACAGGTTGCTGGCGGTCTggctggtggtactggtgaagcccagatggtggaagtaccacaagctgccgcacagattgttgacgtacctgagaacgaggtttcccctgagggcgcagcacagacagaaagcggacgcaagaaagctcgtgctggcggtaagcaaaccttgagtgtagcctgtagctccggt                                                                                                                                  >  1:27856‑M1/1‑129 (MQ=255)`  
                                                                                                                                                                           `gCGTGTATACTTTAAGGAGGTATAAGTTATGGGTAAGAAAGTTAAGAAGGCCGTGAAGAAAGTCACCAAGTCCGTTAAGAAAGTCGTTAAGGAAGGGGCTCGTCCGGTTAAACAGGTTTCTGGCGGTCTAGCTGGTCTGGCTGGTGGTACTGGTGAAGCACAGATGGTGGAAGTACCACAAGCTGCCGCACAGATTGTTGACGTACCTGAGAAAGAGGTTTCCACTGAGGACGAAGCACAGACAGAAAGCGGACGCAAGAAAGCTCGTGCTGGCGGTAAGAAATCCTTGAGTGTAGCCCGt                                                                                                                                  >  1:138035/1‑301 (MQ=255)`  
                                                                                                                                                                           `gCGTGTATACTTTAAGGAGGTATAAGTTATGGGTAAGAAAGTTAAGAAGGCCGTGAAGAAAGTCACCAAGTCCGTTAAGAAAGTCGTTAAGGAAGGGGCTCGTCCGGTTAAACAGGTTGCTGGCGGTCTAGCTGGTCTGGCTGGTGGTACTGGTGAAGCACAGATGGTGGAAGTACCACAAGCTGCCGCACAGATTGTTGACGTACCTGAGAAAGAGGTTTCCACTGAGGACGAAGCACAGACAGAAAGCGGACGCAAGAAAGCTCGTTCTGGCGGTAAGAAATCCTTGAGTGTAGCCCGt                                                                                                                                  >  1:61967/1‑301 (MQ=255)`  
                                                                                                                                                                           `gCGTGTATACTTTAAGGAGGTATAAGTTATGGGTAAGAAAGTTAAGAAGGCCGTGAAGAAAGTCACCAAGTCCGTTAAGAAAGTCGTTAAGGAAGGGGCTAGTCCGGTTAAACAGGTTGCTGGCGGTCTAGCTGGTCTGGCTGGTGGTACTGGTGAAGCACAGATGGTGGAAGTACCACAAGCTGCCGCACAGATTGTTGACGTACCTGAGAAAGAGGTTTCCACTGAGGACGAAGCACAGACAGAAAGCGGACGCAAGAAAGCTCGTGCTGGCGGTAAGAAATCCTTGAGTGTAGCCCGt                                                                                                                                  >  1:271513/1‑301 (MQ=255)`  
                                                                                                                                                                            `cGTGTATACTTTAAGGAGGTATAAGTTATGGGTAAGAAAGTTAAGAAGGCCGTGAAGAAAGTCACCAAGTCCGTTAAGAAAGTCGTTAAGGAAGGGGCTCGTCCGGTTAAACAGGTTGCTGGCGGTCTAGCTGGTCTGGCTGGTGGTACTGGTGAAGCACAGATGGTGGAAGTACCACAAGCTGCCGCACAGATTGTTGACGTACCTGATAAAGAGGTTTCCACTGAGGACGAAGCACAGACAGAAAGCGGACGCAAGAAAGCTCGTGCTGGCGGTAAGAAATCCTTGAGTGTAGCCCGTa                                                                                                                                 >  1:4783/1‑301 (MQ=255)`  
                                                                                                                                                                             `gtgtATACTTTAAGGAGGTATAAGTTAGGGGTAAGAAAGTTAAGAAGGCCGTGAAGAAAGTCACCAAGTCCGTTAAGAAAGTCGTTAAGGAAGGGGCTCGGCCGGTTAAACAGGTGGCTGGCGGTCTagctggtggtactggtgaagcacagatggtggaagtaccacaagctgccgaacagattgttgacgtacctgagaaagaggtttccactgaggacgaagcacagacagaaagcggacgcaagaaagctcgtgctggcggtaagaaatccttgagtgtagcccgtagctccggtgg                                                                                                                                <  1:246161‑M1/301‑175 (MQ=255)`  
                                                                                                                                                                                    `cTTTAAGGAGGTATAAGTTATGGGTAAGAAAGTTAAGAAGGCCGTGAAGAAAGTCACCAAGTCCGTTAAGAAAGTCGTTAAGGAAGGGGCTCGTCCGGTTAAACAGGTTGCTGGCGGTCTggctggtggtactggtgaagcacagatggtggaagtaccacaagctgccgcacagattgttgacgtacctgagaaagaggtttccactgaggacgaagcacagacagaaagcggacgcaagaaagctcgtgctggcggtaagaaatccttgagtgtagcccgtagctccggtggcggtatc                                                                                                                         >  1:309920‑M1/1‑120 (MQ=255)`  
                                                                                                                                                                                           `gaggTATACGTTATGGGTAAGAAAGTTAAGAAGGCCGTGAAGAAAGTCACCAAGTCCGTTAAGAAAGTCGTTAAGGAAGGGGCTAGTCCGGTTAAACAGGTTGCTGGCGGTCTAGCTGGTCTGGCTGGTGGTACTGGTGAAGCACAGATGGTGGAAGTACCACAAGCTGCCGCACAGATTGTTGACGTACCTGAGAAAGAGGTTTCCACTGCGGACGAAGCACAGACAGAAAGCGGACGCAAGAAAGCTCGTGCTGGCGGTAAGAAATCCTTGAGTGTAGCCCGTAGCTCCGGTGGCGGtt                                                                                                                  >  1:164509/1‑300 (MQ=255)`  
                                                                                                                                                                                            `aggTATAAGTTATGGGTAAGAAAGTTAAGAAGGCCGTGAAGAAAGTAACCAAGTCCGTTAAGAAAGTCGTTAAGGAAGGGGCTCGTCCGGTTAAACAGGTTGCTGGCGGTCTGGCTGGTCTGGCTGGTGGTACTGGTGAAGCACAGATGGTGGAAGTACCACAAGCTGCCGCACAGATTGTTGACGTACCTGAGAAAGAGGTTTCCACTGAGGACGAAGCACAGACAGAAAGAGGACGCAAGAAAGCTCGTGCTGGCGGTAAGAAATCCTTGAGTGTAGCCCGTAGCTCCGGTGGCGGTAt                                                                                                                 >  1:312296/1‑301 (MQ=255)`  
                                                                                                                                                                                             `ggTATAAGTTATGGGTAAGAAAGTTAAGAAGGCCGTGAAGAAAGTCACCAAGTCCGTTAAGAAAGTCGTTAAGGAAGGGGCTCGTCCGGTTAAACAGGTTGCTGGCGGTCTggctggtggtactggtgaagcacagatggtggaagtaccacaagctgccgcacagattgttgacgtgcctgagaaagaggtttccactgaggacgaagcacagacagaaagcggacgcaagaaagctcgtgctggcggtaagaaatccttgagtgtagcccgtagctccggtggcggtatcaacatttaa                                                                                                                >  1:214747‑M1/1‑111 (MQ=255)`  
                                                                                                                                                                                             `ggTATAAGTTATGGGTAAGAAAGTTAAGAAGGCCGTGAAGAAAGTCACCAAGTCCGTTAAGAAAGTCGTTAAGGAAGGGGCTCGTCCGGTTAAACAGGTTGCTGGCGGTCTggctggtggtactggtgaagcacagatggtggaagtaccacaagctgccgcacagattgttgacgtacctgagaaagaggtttccactgaggacgaagcacagacagaaagcggacgcaagaaagctcgtgctggcggtaagaaatccttgagtgtagcccgtagctccggtggcggtatcaacatttaa                                                                                                                >  1:103732‑M1/1‑111 (MQ=255)`  
                                                                                                                                                                                             `ggTATAAGTTATGGGTAAGAAAGTTAAGAAGGCCGTGAAGAAAGTCACCAAGTCCGTTAAGAAAGTCGTTAAGGAAGGGGCTCGTCCGGTTAAACAGGTTGCTGGCGGTCTggctggtggtactggtgaagcacagatggtggaagtaccacaagctgccgcacagattgttgacgtacctgagaaagaggtttccactgaggacgaagcacagacagaaagcggacgcaagaaagctcgtgctggcggtaagaaatccttgagtgtagcccgtagctccggtggcggtatcaacatttaa                                                                                                                >  1:169406‑M1/1‑111 (MQ=255)`  
                                                                                                                                                                                             `ggTATAAGTTATGGGTAAGAAAGTTAAGAAGGCCGTGAAGAAAGTCACCAAGTCCGTTAAGAAAGTCGTTAAGGAAGGGGCTCGTCCGGTTAAACAGGTTGCTGGCGGTCTggctggtggtactggtgaagcacagatggtggaagtaccacaagctgccgcacagattgttgacgtacctgagaaagaggtttccactgaggacgaagcacagacagaaagcggacgcaagaaagctcgtgctggcggtaagaaatccttgagtgtagcccgtagctcaggtggcggtatcaccattctg                                                                                                                >  1:260505‑M1/1‑111 (MQ=255)`  
                                                                                                                                                                                             `ggTATAAGTTATGGGTAAGAAAGTTAAGAAGGCCGTGAAGAAAGTCACCAAGTCCGTTAAGAAAGTCGTTAAGGAAGGGGCTCGTCCGGTTAAACAGGTTGCTGGCGGTCTggctggtggtactggtgaagcacagatggtggaagtaccacaagctgccgcacagattgttgacgtacctgagaaagaggtttccactgaggacgaagcacagacagaaagcggacgcaagaaagctcgtgctggcggtaagaaatccttgagtgtagccagtagctccggtggcggtatcaacatttaa                                                                                                                >  1:218851‑M1/1‑111 (MQ=255)`  
                                                                                                                                                                                              `gTATAAGTTATGGGTAAGAAAGTTAAGAAGGCCGTGAAGAAAGTCACCAATTCCGTTAAGAAAGTCGTTAAGTAAGGGGCTCGTCCGGTTAAACAGGTTGCTGGCGGTCTggctggtggtactggtgacgcacagatggtggaagtaccacaagctgccgcacagattgttgacgtacctgagaaagaggtttccactgaggacgaagcacagacagaaagcggacgcaagaaagctcgtgctggcggtaagaaatccttgagtgtagcccgtagctccggtggcggtatcaacatttaat                                                                                                               >  1:110867‑M1/1‑110 (MQ=255)`  
                                                                                                                                                                                              `gTATAAGTTATGGGTAAGAAAGTTAAGAAGGCCGTGAAGAAAGTCACCAAGTCCGTTAAGAAAGTCGTTAAGGAAGGGGCTCGTCCGGTTAAACAGGTTTCTGGCGGTCTAGCTGGTCTGGCTGGTGGTACTGGTGAAGCACAGATGGTGGAAGTACCACAAGCTGCCGCACAGATTGTTGACGTACCTGAGAAAGAGGTTTCCACTGAGGACGAAGCACAGACAGAAAGCGGACGCAAGAAAGCTCGTGCTGGCGGTAAGAAATCCTTGAGTGTAGCCCGTAGCTCCGGTGGCGGTATCa                                                                                                               >  1:291464/1‑301 (MQ=255)`  
                                                                                                                                                                                              `gTATAAGTTATGGGTAAGAAAGTTAAGAAGGCCGTGAAGAAAGTCACCAAGTCCGTTAAGAAAGTCGTTAAGGAAGGGGCTCGTCCGGTTAAACAGGTTGCTGGCGGTCTggctggtggtactggtgaagcacagatggtggaagtaccacaagctgccgcacagattgttgacgtacctgagaaagaggtttccactgaggacgaagcacagacagaaagcggacgcaagaaagctcgtgctggcggtaagaaatccttgagtgtagcccgtagctccggtggcggtatcaacatttaat                                                                                                               >  1:220807‑M1/1‑110 (MQ=255)`  
                                                                                                                                                                                               `tataAGTTGTGGGTAAGTAAATCAAGAAGGCCGTGAAGAAAGTCACCAAGTCCGTTAAGAAAGTCGTTAAGGAAGGGGATCGTGCGGTTAGACAGGTTGCTGGCGGTCTggctggttgtactggtgaagcacagatggtggaagtaccacaagctgccgcacagattgttgacgtacctgagaaagaggtttccactgaggacgaagcacagacagaaagcggacgcaagaaagctcgtgctggcggtaagaaatccttgagtgtagcccgtagctccggtggcggtatcaacatttaatc                                                                                                              <  1:287202‑M1/301‑193 (MQ=255)`  
                                                                                                                                                                                                   `aGTTATTGGTAAGAAAGTTAAGAAGGCCGTGAAGAAAGTCACCAAGTCCGTTAAGAAAGTCGTTAAGGAAGGGGCTCGTCCGGTTAAACAGGTTTCTGGCGGTCTAGCTGGTCTGGCTGGTGGTACTGGTGAAGCACAGATGGTGGAAGTACCACAAGCTGCCGCACAGATTGTTGACGTACCTGAGAAAGAGGTTTCCACTGAGGACGAAGCACAGACAGAAAGCGGACGCAAGAAAGCTCGTGCTGGCGGTAAGAAATCCTTGAGTGTAGCCCGTAGCTCCGGTGGCGGTATCAACAtt                                                                                                          >  1:229868/1‑301 (MQ=255)`  
                                                                                                                                                                                                    `gTTATGGGTAAGAAAGTTAAGAAGGCCGTGAAGAAAGTCACCAAGTCCGTTAAGAAAGTCGTTACGGAAGTGGCTCGTCCGGTTAAACAGGTTGCTGGCGGTTTggctggtggtactggtgaagcacagatggtggaagtaccacaagctgccgcacagattgttgacgtacctgagaacgaggtttccactgaggacgaagcacagacagaacgcggccgcaaacaagctcgtgctggcggtaagaaatccttgagtgtagcacgttactccggtggcggtatccacatttactcaggag                                                                                                         >  1:452‑M1/1‑104 (MQ=255)`  
                                                                                                                                                                                                       `aTGGGTAAGAAAGTTAAGAAGGCCGTGAAGAAAGTCACCAAGTCCGTTAAGAAAGTCGTTAAGGAAGGGGCTCGTCCGGTTAAACAGGTTTCTGGCGGTCTAGCTGGTCTGGCTGGTGGTACTGGTGAAGCACAGATGGTGGAAGTACCACAAGCTGCCGCACAGATTGTTGACGTACCTGAGAAAGAGGTTTCCACTGAGGACGAAGCACAGACAGAAAGCGGACGCAAGAAAGCTCGTGCTGGCTGTAAGAAATCCTTGAGTGTAGCCCGTAGCTCCGGTGGCGGTATCAACATTTAAt                                                                                                      <  1:167222/301‑1 (MQ=255)`  
                                                                                                                                                                                                                   `gTTAAGAAGGCCGTGAAGAAAGTCACCAAGTCCGTTAAGAAAGTCGTTAAGGAAGGGGCTCGTCCGGTTAAACAGGTTGCTGGCGGTCTggctggtggtactggtgaagcacagatggtggaagtaccacaagctgccgcacagattgttgacgtacctgagaaagaggtttccactgaggacgaagcacagacagaaagcggacgcaagaaagctcgtgctggcggtaagaaatccttgagtgtagcccgtagctccggtggcggtatcaacatttaatcaggaggttatcgtggaagac                                                                                          >  1:251861‑M1/1‑89 (MQ=255)`  
                                                                                                                                                                                                                      `aagaagGCCGTGAAGAAAGTCACCAAGTCCGTTAAGAAAGTCGTTAAGGAAGGGGCTCGTCCGGTTAAACAGGTTGCTGGCGGTCTggctggtggtactggtgaagcacagatggtggaagtacaacaagctgccgcacagattgttgacgtacctgagaaagaggtttccactgaggacgaagcacagacagaaagcggacgcaagaaagctcgtgctggcggtaagaaatccttgagtgtagcccgtagctccggtggcggtatcaacatttaatcaggaggttatcgtggaagactgc                                                                                       <  1:96397‑M1/301‑216 (MQ=255)`  
                                                                                                                                                                                                                             `ccGTGAAGAAAGTCACCAAGTCCGTTAAGAAAGTCGTTAAGGAAGGGGCTCGTCCGGTTAAACAGGTTGCTGGCGGTCTggctggtggtactggtgaagcacagatggtggaagtaccacaagctgccgcacagattgttgacgtacctgagaaagaggtttccactgaggacgaagcacagacagaaagcggacgcaagaaagctcgtgctggcggtaagaaatccttgagtgtagcccgtagctccggtggcggtatcaacatttaatcaggaggttatcgtggaagactgcattgaat                                                                                <  1:266974‑M1/301‑223 (MQ=255)`  
                                                                                                                                                                                                                             `ccGTGAAGAAAGTCACCAAGTCCGTTAAGAAAGTCGTTAAGGAAGGGGCTCGTCCGGTTAAACAGGTTGCTGGCGGTCTggctggtggtactggtgaagcacagatggtggaagtaccacaagctgccgcacagattgttgacgtacctgagaaagaggtttccactgaggacgaagcacagacagaaagcggacgcaagaaagctcgtgctggcggtaagaaatccttgagtgtagcccgtagctccggtggcggtatcaacatttaatcaggaggttatcgtggaagactgcattgaat                                                                                <  1:136049‑M1/301‑223 (MQ=255)`  
                                                                                                                                                                                                                                  `aagaaAGTCACCAAGTCCGTTAAGAAAGTCGTTAAGGAAGGGGCTCGTCCGGTTAAACAGGTTGCTGGCGGTCTggcttgtggtactggtgaagcacagatggtggaagtaccacaagctgccgcacagattgttgacgtacctgagaaagaggtttccactgaggacgaagcacagacagaaagcggacgcaagaaagctcgtgctggcggtaagaaatccttgagtgtagcccgtagctccggtggcggtatcaacatttaatcaggaggttatcgtggaagactgcattgaatggacc                                                                           <  1:69539‑M1/301‑228 (MQ=255)`  
                                                                                                                                                                                                                                      `aaGTCACCAGGGCCGGGAAGAAAGTCGTTAAGGAAGGGGCTCGGACGGGTAAACAGGTTGCTGGCGGTGTAGCTGGTCGGGCGGGTGGTACTGGTGAAGCACAGATGGTGGAAGTACCAAAAGCTGCCGCACAGATTGTTGACGTACCTGAGAAAGAGGTTTCCACTGATGACGAAGCACAGACAGAAAGCGGACGCAAGAAAGCTCGTGCTGGCGGTAAGAAATCCTTGAGTGTAGCCCGTAGCTCCGGTGGCGGTATCAACATTGAATCAGGAGGTGATCGTGGAAGACTGCATTGAAt                                                                       <  1:77062/301‑1 (MQ=255)`  
                                                                                                                                                                                                                                          `cACCAAGTCCGTTAAGAAAGTCGTTAAGGAAGGGGCTCGTCCGGTTAAACAGGTTGCTGGCGGTCTggctggtggtactggtgaagcacagatggtggaagtaccacaagctgccgcacagattgttgacgtacctgagaaagaggtttccactgaggacgaagcacagacagaaagcggacgcaagaaagctcgtgctggcggtaagaaatccttgagtgtagcccgtagctccggtggcggtatcaacatttaatcaggaggttatcgtggaagactgcattgaatggaccggaggtgt                                                                   >  1:225218‑M1/1‑66 (MQ=255)`  
                                                                                                                                                                                                                                          `cACCAAGTCCGTTAAGAAAGTCGTTAAGGAAGGGGCTCGTCCGGTTAAACAGGTTGCTGGCGGTCTAGCTGGTCTGGCTGGTGGTACTGGTGAAGCACAGATGGTGGAAGTACCACAAGCTGCCGCACAGATTGTTGACGTACCTGAGAAAGAGGTTTCCACTGAGGACGAAGCACAGACAGAAAGCGGACGCAAGAAAGCTCGTGCTGGCTGTAAGAAATCCTTGAGTGTAGCCCGTAGCTCCGGTGGCGGTATCAACATTTAATCAGGAGGTTATCGTGGAAGACTGCATTGAATGTAc                                                                   >  1:148868/1‑301 (MQ=255)`  
                                                                                                                                                                                                                                               `aGTCCGTTCAGAAAGTCGTTAAGGAGGGGGCGCGTCCGGTTAAACATGTTTCTGGCGGTCTAGCTGGTCTGGCTGGTGGTACTGGTGAAGCACAGATGGTGGAAGTACCACAAGCTGCCGCACAGATTGTTGACGTACCTGAGAAAGAGGTTTCCACTTAGGACGAAGCACAGACAGAAAGCGGACGCAAGAAAGCTCGTGCTGGCGGTAAGAAATCCTTGAGTGTAGCCCGTAGCTCCGGTGGCGGTATCAACATTTAATCAGGAGGTTATCGTGGAAGACTGCATTGAATGGACCGGAg                                                              <  1:187915/301‑1 (MQ=255)`  
                                                                                                                                                                                                                                                        `aGAAAGTCGTTAAGGAAGGGGCTCGTCCGGTTAAACAGGTTTCTGGCGGTCTAGCTGGTCTGGCTGGTGGTACTGGTGAAGCACAGATGGTGGAAGTACCACAAGCTGCCGAACAGATTGTTGACGTACCTGAGAAAGAGGTTTCCACTGAGGACGAAGCACAGACAGAAAGCGGACGCAAGAAAGCTCGTGCTGGCGGTAAGAAATCCTTGATTGTAGCCCGTAGCTCCGGTGGCGGTATCAACATTTAATCAGGAGGTTATCGTGGAAGACTGCATTGAATGGACCGGAGGTGTCAAct                                                     <  1:323284/301‑1 (MQ=255)`  
                                                                                                                                                                                                                                                        `aGAAAGTCGTTAAGGAAGGGGCTCGTCCGGTTAAACAGGTTGCTGGCGGTCTggctggtggtactggtgaagcacagatggtggaagtaccacaagctgccgcacagattgttgacgtacctgagaaagaggtttccactgaggacgaagcacagacagaaagcggacgcaagaaagctcgtgctggcggtaagaaatccttgagtgtagcccgtagctccggtggcggtatcaacatttaatcaggaggttatcgtggaagactgcattgaatggaccggaggtgtcaactctaagggtt                                                     >  1:113625‑M1/1‑52 (MQ=255)`  
                                                                                                                                                                                                                                                        `aGAAAGTCGTTAAGGAAGGGGCTCGTCCGGTTAAACAGGTTGCTGGCGGTCTggctggtggtactggtgaagcacagatggtggaagtaccacaagctgccgcacagattgttgacgtacctgagaaagaggtttccactgaggacgaagcacagacagaaagcggacgcaagaaagctcgtgctggcggtaagaaatccttgagtgtagcccgtagctccggtggcggtatcaacatttaatcaggaggttatcgtggaagactgcattgaatggaccggaggtgtcaactctaagggtt                                                     <  1:302751‑M1/301‑250 (MQ=255)`  
                                                                                                                                                                                                                                                             `ggcgtTAAGGAAGGGGCTCGTCCGGTTAAACAGGTTGCTGGCGGTCTggatggtggtactggtgaagcacagatggtggaagtaccacaagctgccgcacagattgttgacgtacctgagaaagaggtttccactgaggacgaagcacagacagaaagcggacgcaagaaagctcgtgctggcggtaagaaatccttgagtgtagcccgtagctccggtggaggtatcaacatttaatcaggaggttatcgtggaagactgcattgaatggaccggaggtgtcaactctaagggttatggt                                                <  1:193038‑M1/299‑255 (MQ=255)`  
                                                                                                                                                                                                                                                             `gTCGTTAAGGAAGGGGCTCGTCCGGTTAAACAGGTTGCTGGCGGTCTggctggtggtactggtgaagcacagatggtggaagtaccacaagctgccgcacagattgttgacgtacctgagaaagaggtttccactgaggacgaagcacagacagaaagcggacgcaagaaagctcgtgctggcggtaagaaatccttgagtgtagcccgtagctccggtggcggtataaacatttaatcaggaggttatagtggaagactgcattgaatggaccggaggtgtcaaatctaaggggtatggg                                                >  1:185375‑M1/1‑47 (MQ=255)`  
                                                                                                                                                                                                                                                             `gTCGTTAAGGAAGGGGCTCGTCCGGTTAAACAGGTTGCTGGCGGTATggctggtggtactggtgaagcacagatggtggaagtaccacaagctgccgcacagattgttgacgtacctgagaaagaggtttccactgaggacgaagcacagacagaaagcggacgcaagaaagctcgtgctggcggtaagaaatccttgagtgtagcccgtagctccggtggcggtatcaacatttaatcaggaggttatcgtggaagactgcattgaatggaccggaggtgtcaactctaagggttatggt                                                <  1:133093‑M1/301‑255 (MQ=255)`  
                                                                                                                                                                                                                                                             `gTCGGTAAGGAAGGGGCTCGTCCGGTTAAACAGGTTGCTGGCGGTCTggctggtggtactggtgaagcacagatggtggaagtaccacaagctgccgcacagattgttgacgtacctgagaaagaggtttccactgaggacgaagcacagacagaaagcggacgcaagaaagctcgtgctggcggtaagaaatccttgagtgtagcccgtagctccggtggcggtatcaacatttaatcaggaggttatcgtggaagactgcattgaatggaccggaggtgtcaactctaagggttatggt                                                <  1:39298‑M1/301‑255 (MQ=255)`  
                                                                                                                                                                                                                                                                      `gaaggGGCTCGTCCGGTTAAACAGGTTTCTGGCGGTCTAGCTGGTCTGGCTGGTGGTACTGGTGAAGCACAGATGGTGGAAGTACCACAAGCTGCCGCACAGATTGTTGACGTACCTGAGAAAGAGGTTTCCACTGAAGACGAAGCACAGACAGAAAGCGGACGCAAGAAAGCTCGTGCTGGCGGTAAGAAATCCTTGAGTGTAGCCCGTAGCTCCGGTTGCGGTATCAACATTTAATCAGGAGGTTATCGTGGAAGACTGCATTGAATGGACCGGAGGTGTCAACTCTAAGGGTTATGGt                                       >  1:140636/1‑301 (MQ=255)`  
                                                                                                                                                                                                                                                                        `aggTGCTCGTCCGGTTAACCAGGTTGCTGGCGGTTTggctggtggtactggtgaagcacagatggtggaagtaccacaagctgccgcacagattgttgacgtacctgagaaagaggtttccactgaggacgaagcacagacagaaagcggaagcaagaaagctcgtgctggcggtaagaaatccttgagtgtagcccgtagctccggtggcggtatcaacatttaatcaggaggttatcgtggaagactgcattgaatggaccggaggtgtcaactctaagggttatggtcgtaagtgggt                                     <  1:24295‑M1/301‑266 (MQ=255)`  
                                                                                                                                                                                                                                                                                       `tAAACAGGTTGCTGGCGGTCTggctggtggtactggtgaagcacagatggtggaagtaccacaagctgccgcacagattgttgacgtaccttagaaagaggtttccactgaggacgaatcacagacatatctcggacgcaactaagctcgtgctggcggtatgaaatccttgtgtgtagcccgtagctccggtggcggtctcaacatttaatcaggaggttatcgtggaagactgcattgaatgtaccggaggtgtcaactctaagggttatggtcgtaagtgggttaatggtaacctttt                      >  1:100102‑M1/1‑21 (MQ=255)`  
                                                                                                                                                                                                                                                                                          `gagggttgggggggggttggatggtgggactggtgaagcaaagatggtggaagtaccacaagctggcgcacagattgttgacgtacatgagaaagaggtttcaactgaggacgaagcacagacagaaagcggaagcaagaaagctcgtgctggcggtaagaaatccttgagtgtagcccgtagctccggtggcggtataaacatttaagcaggaggttatcgtggaagaatgcattgaatggaccggaggtgtcaactctaagggttatggtcgtaagtgggttaatggtaaacttgtgac                   <  1:175128‑M1/284‑284 (MQ=255)`  
                                                                                                                                                                                                                                                                                                    `ggCGGTCTggctggtggtactggtgaagcacagatggtggaagtaccacaagctgccgcacagattgttgacgtacctgagaaagaggtttccactgaggacgaagcacagacagaaagcggacgcaagaaagctcgtgctggcggtaagaaatccttgagtgtagcccgtagctccggtggcggtatcaacatttaatcaggaggttatcgtggaagactgcattgaatggaccggaggtgtaaactctaagggttatggtcgtaagtgggttaatggtaaacttgtgactccacatagg         <  1:197587‑M1/301‑294 (MQ=255)`  

                                                                                                                                                                                                                                                                                                           `|`                                                                                                                                                                                                                                                                                                              
`AAGCTGTGACATTCACCACAAAGGAGACATTCTGGATGTAACGTCCGTTGTCATTAATCCTGAGGCAGACTCTAAGGGCTTACAGCGATTCCTAGCGAAACGCTTTAAGTACCTTGCGGAACTCCACGATTGCGATTGGGTGTCTCGTTGTAAGCATGAAGGCGAGACAATGCGTGTATACTTTAAGGAGGTATAAGTTATGGGTAAGAAAGTTAAGAAGGCCGTGAAGAAAGTCACCAAGTCCGTTAAGAAAGTCGTTAAGGAAGGGGCTCGTCCGGTTAAACAGGTTGCTGGCGGTCTAGCTGGTCTGGCTGGTGGTACTGGTGAAGCACAGATGGTGGAAGTACCACAAGCTGCCGCACAGATTGTTGACGTACCTGAGAAAGAGGTTTCCACTGAGGACGAAGCACAGACAGAAAGCGGACGCAAGAAAGCTCGTGCTGGCGGTAAGAAATCCTTGAGTGTAGCCCGTAGCTCCGGTGGCGGTATCAACATTTAATCAGGAGGTTATCGTGGAAGACTGCATTGAATGGACCGGAGGTGTCAACTCTAAGGGTTATGGTCGTAAGTGGGTTAATGGTAAACTTGTGACTCCACATA  >  NC_001604.1/19336‑19935`

  

Alignment Legend

Aligned base mismatch/match (shaded by quality score): `ATCG/ATCG` `< 3 ≤` `ATCG/ATCG` `< 11 ≤` `ATCG/ATCG` `< 24 ≤` `ATCG/ATCG` `< 35 ≤` `ATCG/ATCG` `< 38 ≤` `ATCG/ATCG`

Unaligned base: `atcg`    Masked matching base: `atcg`    Alignment gap: `‑`    Deleted base: `‑`

Reads not counted as support for junction

read\_name Not counted due to insufficient overlap past the breakpoint.

read\_name Not counted due to not crossing MOB target site duplication.
